# Bexagliflozin on Renal Outcomes in Type 2 Diabetes Mellitus: A Systematic Review and Meta-Analysis of Randomized Controlled Trials

**DOI:** 10.7759/cureus.82458

**Published:** 2025-04-17

**Authors:** Marília Gobbo, Renan Y Ura Sudo, Tanize L Milbradt, Isabel Cristina Reinheimer, Matthew Min, Carlos E Poli-de-Figueiredo

**Affiliations:** 1 Medical School, Pontifical Catholic University of Rio Grande do Sul, Porto Alegre, BRA; 2 Medicine, Federal University of Grande Dourados, Dourados, BRA; 3 Medicine, Federal University of Santa Maria, Santa Maria, BRA; 4 Nephrology, Pontifical Catholic University of Rio Grande do Sul, Porto Alegre, BRA; 5 Primary Care Internal Medicine, University of Connecticut, Storrs, USA

**Keywords:** bexagliflozin, diabetes, kidney function, renal outcomes, sglt2 inhibitors, type 2 diabetes mellitus

## Abstract

Sodium-glucose cotransporter-2 (SGLT2) inhibitors are important for treating type 2 diabetes mellitus (T2DM). However, it remains unclear whether the newest SGLT2 inhibitor, bexagliflozin, provides benefits for renal- or urinary-related outcomes. The ClinicalTrials.gov, PubMed, Embase, and Cochrane databases were searched for randomized controlled trials. Using R software version 4.3.1 (R Foundation for Statistical Computing, Vienna, Austria), a random-effects model was employed to compute mean differences (MD) and risk ratios for continuous and binary endpoints. The Grading of Recommendations, Assessment, Development, and Evaluation (GRADE) approach was used to rate the certainty of evidence. The International Prospective Register of Systematic Reviews (PROSPERO) identification number is CRD42023478336. Nine studies involving 4,352 patients were included. Over a follow-up that ranged from 12 to 96 weeks, patients taking bexagliflozin showed no changes in serum creatinine levels (MD: 0.05 mg/dL; 95% CI: -0.06 to 0.15; p = 0.35) or estimated glomerular filtration rate (MD: -0.43 mL/min/1.73 m^²^; 95% CI: -6.92 to 6.06; p = 0.89). However, there was a significant reduction in systolic blood pressure in the treatment group (MD: -4.2 mmHg; 95% CI: -5.6 to -2.8; p < 0.01). In large placebo-controlled trials, we observed no beneficial effect of bexagliflozin on kidney function, as described with other SGLT2 inhibitors.

## Introduction and background

Diabetes is a leading cause of death worldwide, responsible for 6.7 million fatalities in 2021 and establishing itself as a major risk factor for the development of cardiovascular and kidney diseases. Particularly noteworthy is the widespread prevalence of type 2 diabetes mellitus (T2DM), accounting for over 90% of all cases globally [1-4]. Careful blood glucose control and targeted pharmacological intervention can slow the progression of macrovascular and microvascular complications associated with diabetes and lead to better management of this condition [5,6].

In this context, sodium-glucose cotransporter 2 (SGLT2) inhibitors have emerged as a promising therapeutic alternative by blocking sodium and glucose cotransportation, promoting glucosuria and natriuresis, particularly in hyperglycemic patients. In addition to their antidiabetic effects, they offer cardiovascular and renal benefits by increasing sodium delivery to the macula densa, leading to the constriction of afferent arterioles, which reduces glomerular hypertension and albuminuria, thereby slowing the progression of renal damage [7-10]. Bexagliflozin was the latest SGLT2 inhibitor approved by the Food and Drug Administration (FDA) in 2023. Studies have shown that it reduces glycated hemoglobin (HbA1c) similarly to other SGLT2 inhibitors in patients with T2DM. While sharing the core mechanism of action with other SGLT2 inhibitors, bexagliflozin exhibits distinct pharmacokinetic properties that set it apart. It has a longer half-life, allowing for once-daily dosing with sustained glycemic control. Additionally, unlike some other SGLT2 inhibitors, bexagliflozin demonstrates minimal off-target inhibition of SGLT1, which may contribute to a more favorable side-effect profile [11-19].

Despite the benefits in renal function and long-term albuminuria observed with other gliflozins, such as canagliflozin, dapagliflozin, and empagliflozin [20-23], the effects of bexagliflozin on renal outcomes remain uncertain. Therefore, this systematic review and meta-analysis aim to conduct a comprehensive analysis of clinical trials examining the effect of bexagliflozin on renal outcomes in adults with T2DM compared to placebo or other oral hypoglycemic agents (OHA).

## Review

Methods

Search Strategy and Eligibility Criteria

This systematic review and meta-analysis follow the recommendations of the Cochrane Collaboration and are reported in accordance with the guidelines outlined in the Preferred Reporting Items for Systematic Reviews and Meta-Analyses (PRISMA) statement. Prior to literature screening, the protocol for this study was recorded prospectively in November 2023 in the International Prospective Register of Systematic Reviews (PROSPERO) database under the identification number CRD42023478336.

We searched the PubMed, Embase, and Cochrane Library electronic databases for studies published from inception to November 2023. To ensure a comprehensive search, we also hand-searched the ClinicalTrials.gov databases. The complete and detailed search strategy used was Bexagliflozin AND (RCT OR randomized OR randomised) AND ("type 2" OR diabetes OR diabetic OR DM OR T2DM).

Reviewers (M.G. and R.S.) independently evaluated titles, abstracts, and full-text articles. Any discrepancies were settled with the help of a third reviewer (T.M.). Studies were deemed eligible if they fulfilled the following inclusion criteria: randomized controlled trial (RCT); comparison of bexagliflozin to matched placebo or other OHA; patients diagnosed with T2DM; and reports on renal outcomes. Studies involving patients with type 1 DM, those lacking a control group, or those published in non-English languages were excluded.

Individual patient-level data were requested from the original authors and sponsors of the included studies. Because the required data were unavailable, a meta-analysis of summary data from published reports was conducted.

Statistical Analysis

Data were extracted independently from the included studies by three authors (R.S., M.G., T.M.) using a standardized structured form. Disagreements were resolved through discussion and, if necessary, by consulting with the senior author (C.E.P.F.).

We synthesized the data using a random-effects meta-analysis with a restricted maximum likelihood estimator. Binary outcomes were calculated using risk ratio (RR), while continuous endpoints were summarized with mean difference (MD). A 95% confidence interval (CI) with a p-value below 0.05 indicated statistical significance. Some heterogeneity due to limited data was anticipated, and analysis was performed by calculating the prediction interval (PI) associated with I-squared statistics, Cochran's Q test, p-value, and Tau. Additionally, subgroup analysis of placebo-controlled trials and meta-regression to evaluate the study-level impact of follow-up time were conducted. All statistical analyses were executed in R software version 4.3.1 (R Foundation for Statistical Computing, Vienna, Austria).

Quality Assessment

Two independent reviewers (T.M. and M.G.) assessed the risk of bias using Cochrane's Risk of Bias 2 (RoB 2) software. Each RCT was assigned a score (low, some concerns, or high) in each of the five domains: selection, performance, detection, attrition, and reporting biases. Disagreements were resolved by consensus. Authors (R.S. and T.M.) adhered to the Grading of Recommendations, Assessment, Development, and Evaluation (GRADE) handbook guidelines to evaluate the certainty level of the evidence, categorizing it from high to very low. The authors utilized the GRADEpro Guideline Development Tool and assessed the following outcomes: serum creatinine concentration and estimated glomerular filtration rate (eGFR).

Results

**Figure 1:**
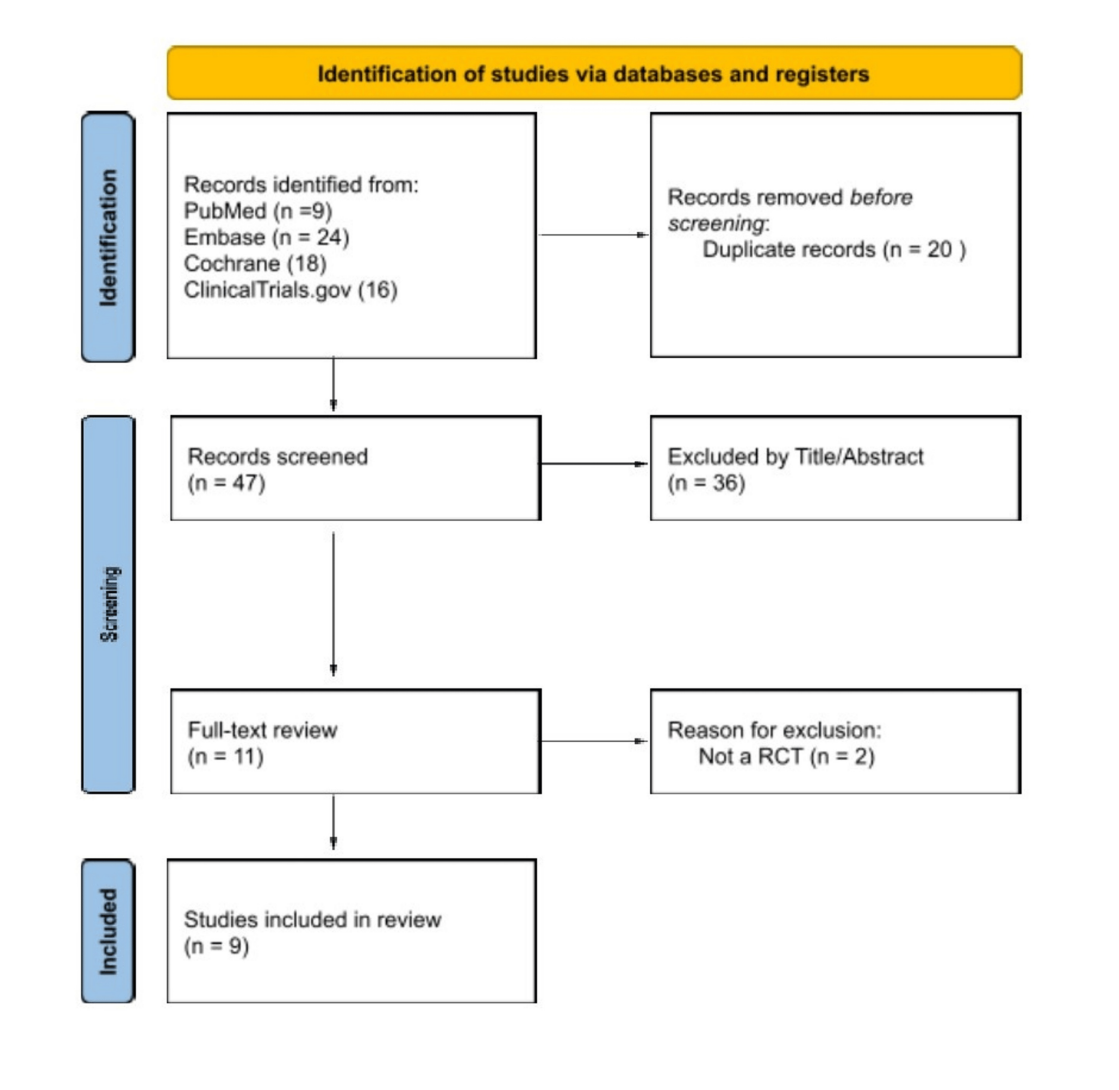
PRISMA 2020 flow diagram. PRISMA 2020 flow diagram for new systematic reviews, which included searches of databases and registers only. PRISMA: Preferred Reporting Items for Systematic Reviews and Meta-Analyses; RCT: randomized controlled trial

As shown in Figure 1, the first search yielded 47 papers. After the initial screening and removal of duplicates, an additional 36 articles were independently excluded by paired reviewers based on title and abstract evaluations. Subsequently, 11 articles underwent full evaluation. In this comprehensive review, two more were excluded due to their non-randomized design. Ultimately, we included nine RCTs, of which three were unpublished National Clinical Trials (NCT), involving 4,352 patients with T2DM [11-19].

Overview of Included Studies

The studies ranged from 2019 to 2023. The interventions across all studies included the administration of oral bexagliflozin, with dosages ranging from 5 mg to 20 mg daily. Therapeutic approaches exhibit variations: six trials used bexagliflozin as a monotherapy [11,12,14,15,18,19], while three studies incorporated metformin as a supplementary medication [13,16,17]. Additionally, five studies compared the intervention to a placebo [11,12,14,18,19], whereas four compared it to other oral antidiabetic drugs [13,15-17]. The follow-up duration ranged from 12 to 96 weeks. The baseline characteristics of all included studies are reported in Table 1. 

**Table 1:** Baseline characteristics of the included studies. Values are presented in means unless otherwise specified. BMI: body mass index; HbA1c: glycated hemoglobin; DB: double-blind; eGFR: estimated glomerular filtration rate; MTF: metformin; NCT: National Clinical Trial; PBO: placebo; RCT: randomized controlled trial

Study/characteristic	Allegretti et al. [11]	Halvorsen et al. [12]	Halvorsen et al. [13]	Halvorsen et al. [14]	Halvorsen et al. [15]	Halvorsen et al. [16]	NCT02769481 [17]	NCT02715258 [18]	NCT02558296 [19]
Year of publication	2019	2019	2019	2019	2022	2023	2021	2021	2021
Intervention	Bexagliflozin	Bexagliflozin	Bexagliflozin and MTF	Bexagliflozin	Bexagliflozin	Bexagliflozin and MTF	Bexagliflozin and MTF	Bexagliflozin	Bexagliflozin
Intervention doses	20 mg	5 mg, 10 mg, and 20 mg	20 mg	20 mg	20 mg	20 mg	20 mg	20 mg	20 mg
Control	PBO	PBO	Sitagliptin and MTF	PBO	Glimepiride	PBO and MTF	Glimepiride and MTF	PBO	PBO
Control doses	Not reported	Not reported	100 mg	Not reported	2-6 mg	Not reported	2 mg, 4 mg, and 6 mg	Not reported	Not reported
Sample size, n (intervention)	157	220	191	145	213	158	213	140	1133
Sample size, n (control)	155	72	193	138	213	159	213	70	567
Follow-up, weeks	24	12	24	96	96	24	96	24	24
Study design	DB RCT	DB RCT	DB RCT	DB RCT	DB RCT	DB RCT	DB RCT	DB RCT	DB RCT
Mean age, years	69.6	59.2	59.4	55.6	59.6	55.8	59.6	55.4	64.4
Male sex, %	62.8	61.6	64.1	41	58.2	61.2	58.2	48.3	69.5
eGFR, ml/min per 1.73 m²	45.1	>60	>60	Not reported	Not reported	>60	Not reported	>60	77.8
BMI, kg/m²	30.2	28.6	31.7	30.1	31.8	29.8	31.8	32.0	32.6
Diabetes duration, years	15.9	6.2	8.7	7.4	8.6	9	Not reported	6.1	14.9
HbA1c, %	7.9	7.7	7.9	Not reported	8.01	8.6	Not reported	8.02	8.3
Systolic blood pressure, mmHg	137	128.4	135.3	127.2	133.8	129.3	133.8	129.2	134
Diastolic blood pressure, mmHg	Not reported	78.5	81	76.9	79.9	81.1	Not reported	Not reported	77

Pooled Analysis of Outcomes

**Figure 2:**
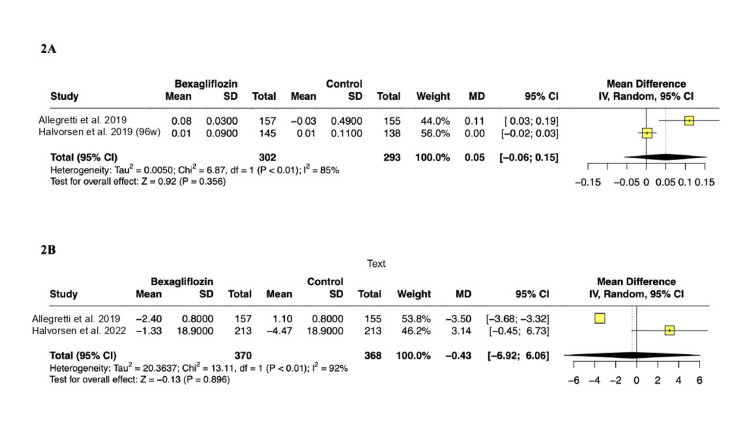
(A) Change in serum creatinine from baseline. (B) Change in eGFR from baseline. References: (A) Allegretti et al. [11]; Halvorsen et al. [14]. (B) Allegretti et al. [11]; Halvorsen et al. [15]. eGFR: estimated glomerular filtration rate; MD: mean difference; SD: standard deviation; CI: confidence interval

Creatinine levels and eGFR:Two RCTs involving 595 patients reported changes in serum creatinine levels among participants. In the pooled analysis, the treatment group showed no significant change in creatinine levels (MD: 0.05 mg/dL; 95% CI: -0.06 to 0.15; p = 0.35; PI: -0.18 to 0.12; Figure 2A). Two RCTs with 738 patients reported changes in eGFR among participants. The pooled analysis indicated no difference between groups (MD: -0.43 mL/min per 1.73 m²; 95% CI: -6.92 to 6.06; p = 0.896; PI: -4.94 to 4.08; Figure 2B).

**Figure 3:**
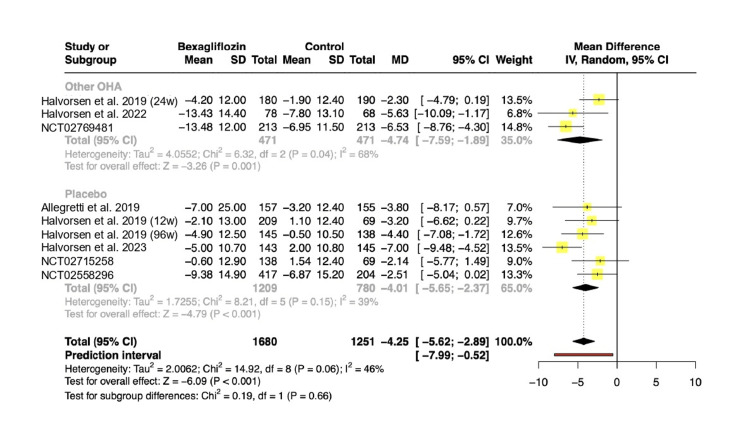
Change in systolic blood pressure from baseline. References: Halvorsen et al. [13]; Halvorsen et al. [15]; NCT02769481 [17]; Allegretti et al. [11]; Halvorsen et al. [12]; Halvorsen et al. [14]; Halvorsen et al. [16]; NCT02715258 [18]; NCT02558296 [19]. OHA: oral hypoglycemic agents; MD: mean difference; SD: standard deviation; CI: confidence interval

Systolic blood pressure (SBP): Nine RCTs encompassing 2,370 patients reported changes in SBP from baseline among participants. The pooled analysis revealed a statistically significant reduction in SBP in the treatment group (MD: -4.25 mmHg; 95% CI: -5.62 to -2.89; p < 0.01; PI: -7.99 to -0.52; Figure 3). There was no statistically significant difference in effect sizes between the subgroups (p = 0.66).

**Figure 4:**
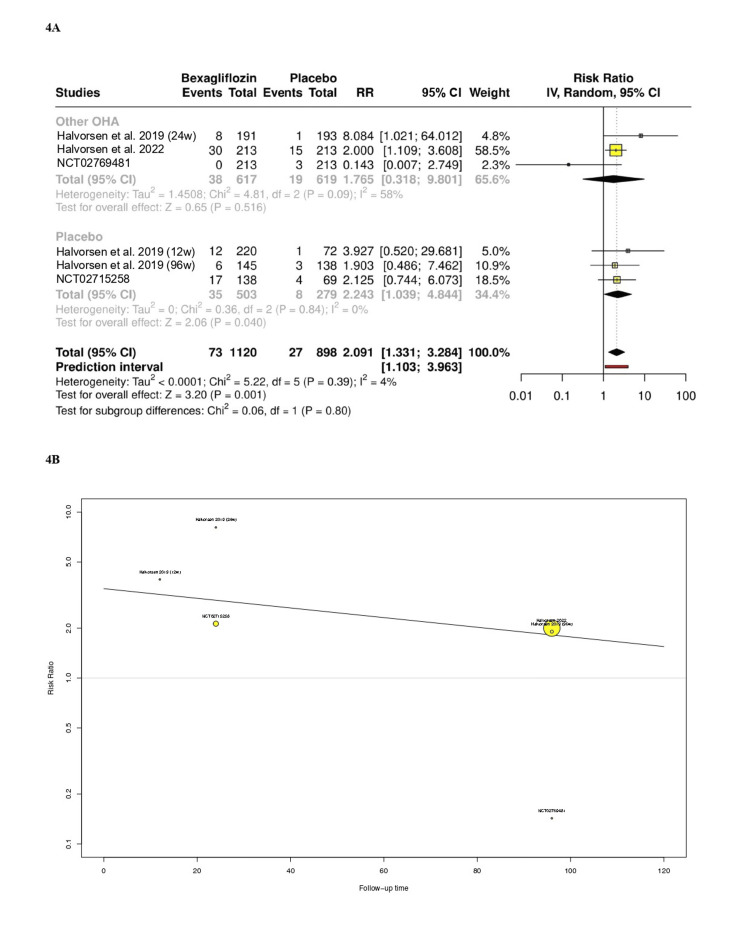
(A) Renal and urinary adverse events reported as a single composite outcome. (B) Meta-regression analysis: impact of follow-up time on renal and urinary adverse events. References: Halvorsen et al. [13]; Halvorsen et al. [15]; NCT02769481 [17]; Halvorsen et al. [12]; Halvorsen et al. [14]; NCT02715258 [18]. OHA: oral hypoglycemic agents; CI: confidence interval

Renal and urinary adverse events: Six RCTs including 2,018 patients reported renal and urinary adverse events as a single composite outcome among participants. In the pooled analysis, the bexagliflozin arm increased the risk of renal or urinary adverse events (RR: 2.09; 95% CI: 1.33 to 3.28; p < 0.01; PI: 1.1 to 3.9; Figure 4A). There was no statistically significant difference in effect sizes between the subgroups of placebo and other oral antidiabetics (p = 0.8). The linear impact of follow-up time on renal and urinary adverse events did not reach statistical significance in the meta-regression analysis, although a trend toward correlation can be observed through eye inspection (p = 0.3; Figure 4B).

**Figure 5:**
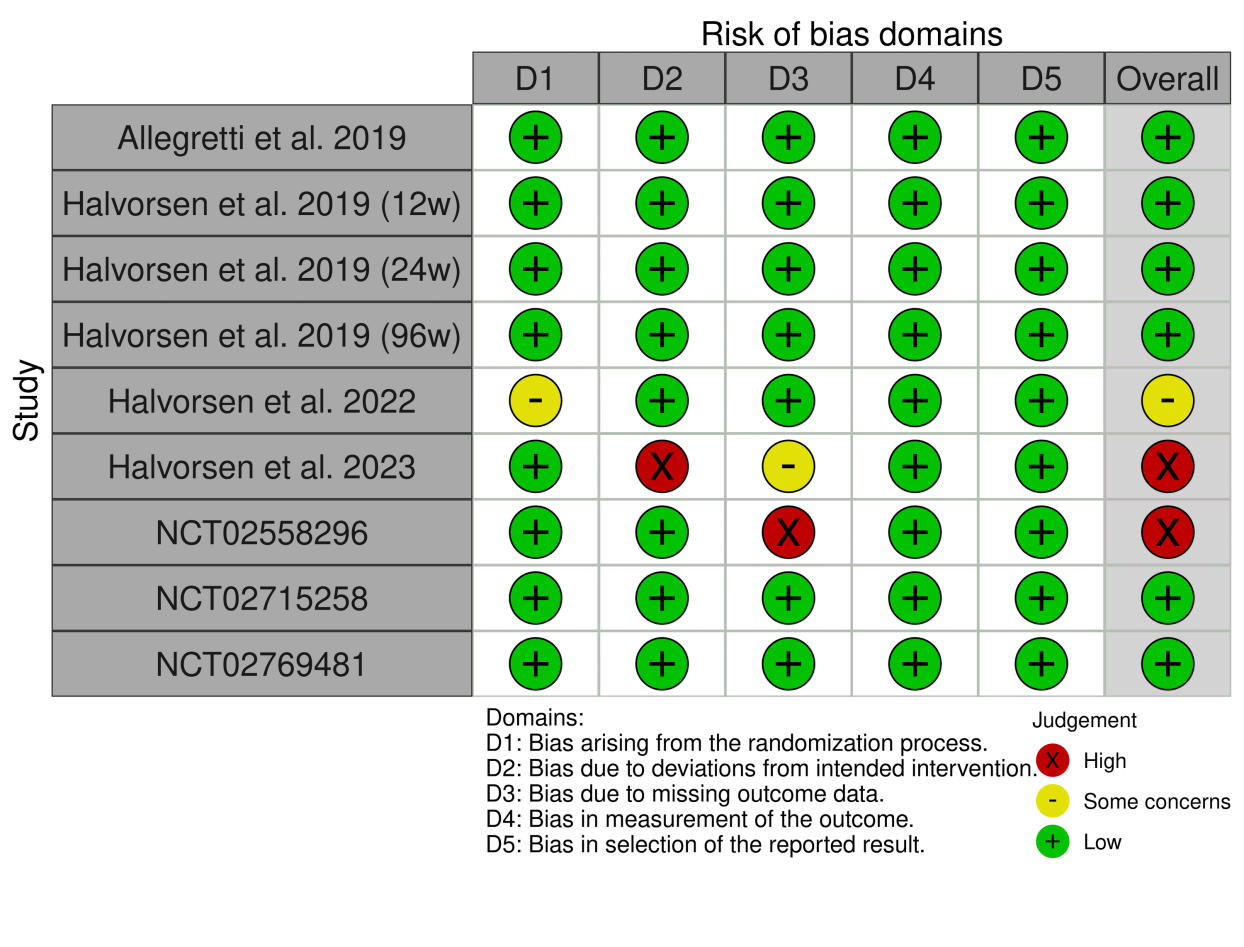
Risk of bias assessment of the included studies. References: Allegretti et al. [11]; Halvorsen et al. [12]; Halvorsen et al. [13]; Halvorsen et al. [14]; Halvorsen et al. [15]; Halvorsen et al. [16]; NCT02769481 [17]; NCT02715258 [18]; NCT02558296 [19].

Risk of bias assessment: Figure 5 outlines a comprehensive assessment of each RCT across all five domains of risk of bias. Among the included studies, one of the studies by Halvorsen et al. [16] and the NCT02558296 [19] were identified as being at high risk of bias due to missing outcome data, while the 2022 study by Halvorsen et al. [15] was categorized as having some concerns, primarily related to the randomization process.

Certainty of evidence and GRADE assessment: The overall certainty of the evidence regarding the examined outcomes was downgraded in specific cases, depending on the severity of imprecision and bias effects. Appendix 1 includes a comprehensive GRADE assessment and a summary of findings.

Discussion

In this systematic review and meta-analysis of nine RCTs involving 4,352 patients, bexagliflozin was compared to either a placebo or OHA in individuals with T2DM. A statistically significant difference was observed between the two groups in SBP and renal and urinary adverse events as a single composite outcome; however, there was no significant difference in eGFR and serum creatinine levels.

Current guidelines recommend using SGLT2 inhibitors in patients with an eGFR above 30 mL/min/1.73 m² or higher, regardless of glucose levels. This recommendation is based on the observed benefits in composite cardiovascular and renal outcomes in large placebo-controlled trials [20,21,23,24] and a meta-analysis [25]. However, the results of the pooled analysis indicated no changes in creatinine and eGFR from baseline compared to the control group. It's important to note that only one study was conducted in the context of chronic renal failure [9], which reported a slight increase in serum creatinine concentration and a decrease in eGFR. These findings raise questions about whether bexagliflozin would have any positive renal effects, as benefits on renal function were not consistently observed across the various studies [9,15,16].

Trials examining SGLT2 inhibitors, such as canagliflozin, dapagliflozin, and empagliflozin, have shown consistent benefits in slowing the progressive decline of renal function and reducing albuminuria [20,22,23]. In contrast, bexagliflozin was linked to an increase in renal adverse events in individual studies and showed no benefits for renal function after initial administration. This difference may be related to the shorter follow-up duration and smaller cohort size compared to larger SGLT2 inhibitor trials.

The Effect of Sotagliflozin on Cardiovascular and Renal Events in Patients with Type 2 Diabetes and Moderate Renal Impairment Who Are at Cardiovascular Risk (SCORED) [26] and Effect of Sotagliflozin on Cardiovascular Events in Patients with Type 2 Diabetes Post Worsening Heart Failure (SOLOIST-WHF) [27] trials did not demonstrate differences in renal outcomes with sotagliflozin compared to placebo. However, these trials were prematurely halted due to a lack of funding and were limited to a mean duration of nine and 16 months. Addressing the significance of extended follow-up, the Kidney Outcomes Associated With Use of SGLT2 Inhibitors in Real-World Clinical Practice (CVD-REAL III) [28], a propensity-matching observational cohort of 35,561 patients, highlighted the renal benefits of gliflozins in diabetic patients, which became evident only after 12 months of follow-up. Similarly, the Canagliflozin and Renal Events in Diabetes with Established Nephropathy Clinical Evaluation (CREDENCE) [21] and Dapagliflozin and Prevention of Adverse Outcomes in Chronic Kidney Disease (DAPA-CKD) [22] studies noted a lower risk of progression of chronic kidney disease with a beneficial effect on eGFR, compared to placebo, only after 12 months of follow-up. The Empagliflozin Outcome Trial in Patients with Chronic Heart Failure and a Reduced Ejection Fraction (EMPEROR-Reduced) [29], which compared empagliflozin to placebo, observed improved eGFR levels after 100 weeks of randomization. In the Dapagliflozin and Prevention of Adverse Outcomes in Heart Failure (DAPA-HF)/Renal [30], the benefit to renal function in diabetic patients became apparent only after 720 days of randomization. The Evaluation of Ertugliflozin Efficacy and Safety Cardiovascular Outcomes Trial (VERTIS-CV) [31] noted a crossover between ertugliflozin and the placebo only after 36-48 weeks of randomization. Remarkably, the Dapagliflozin Effect on Cardiovascular Events-Thrombolysis in Myocardial Infarction 58 (DECLARE-TIMI 58) [32], which compared dapagliflozin to placebo, observed a crossover in renal composite outcomes after 540 days of randomization. Likewise, in a pooled analysis on SGLT2 inhibitors' impact on renal outcomes at the longest follow-ups, the risk of progressive kidney disease was lower in the treatment group regardless of baseline eGFR values [25]. Therefore, the potential detrimental and neutral effects of bexagliflozin on renal outcomes and renal function, respectively, raise questions about its impact during prolonged follow-up periods.

Regular screening for urine albumin-to-creatinine ratio (UACR) is recommended for individuals with T2DM to detect kidney disease in its early stages [33], as its elevation is often an early sign of diabetic nephropathy. Other SGLT2 inhibitors have demonstrated a reduction in UACR levels that is associated with slowing the progression of kidney disease. For instance, the DECLARE-TIMI 58 trial [32] showed an improved UACR from baseline across all eGFR categories when dapagliflozin was compared to placebo over a four-year follow-up period. Additionally, a post hoc analysis of the CREDENCE trial [21] indicated an early 30% reduction in albuminuria with canagliflozin during a 26-week follow-up, which correlated with fewer kidney outcomes. However, it is noteworthy that the studies included in this meta-analysis did not provide explicit information regarding changes in UACR levels. The lack of consistent reporting of UACR outcomes across these studies presents a significant data gap in our assessment of the impact of these interventions on renal health. Several factors may contribute to the development of elevated UACR in T2DM, including prolonged high blood glucose levels and hypertension. Further studies on the effect of bexagliflozin on UACR levels are warranted.

Among the included trials, bexagliflozin was significantly associated with a reduction in HbA1c levels, reflecting the effects on glucose parameters similar to other SGLT2 inhibitors [34]. However, the underlying mechanisms of the renal outcomes related to SGLT2 inhibitors are not fully understood. Increasing evidence suggests that hemodynamic factors, such as reduced blood pressure, play a key role, without a clear link between glucose levels and kidney events [24]. In our pooled analysis, bexagliflozin was associated with a four-point mean reduction in SBP. Although there was no data to pool diastolic blood pressure (DBP) values, individual studies [9,13,14] reported a nonsignificant difference between groups. Similarly, an updated meta-analysis [34] indicated a notable reduction in SBP levels, which was not linked to worsening renal function or increased renal adverse events.

The strengths of our study include the large sample size derived exclusively from RCTs and the focus on a recently FDA-approved drug, providing valuable insights in an area where evidence from cohort studies is currently limited. However, limitations should be noted. First, the short follow-up duration in the included studies limits the ability to fully assess the long-term effects of bexagliflozin on renal function and related outcomes. This temporal constraint likely precludes the observation of meaningful changes in key renal endpoints, such as sustained declines in eGFR, initiation of dialysis, or progression to kidney failure. Second, limited availability of outcome data prevented a more granular stratification of results, particularly by baseline eGFR levels. Stratified analyses could have provided deeper insights into the efficacy and safety of bexagliflozin in specific subgroups, such as patients with varying degrees of chronic kidney disease, or using different anti-hypertensive drugs. Third, the meta-analysis highlights a significant increase in the risk of a composite outcome encompassing renal and urinary adverse events. However, this approach, adopted from the included studies, raises interpretative challenges, as it combines fundamentally distinct clinical entities. Grouping renal and urinary outcomes together may obscure the specific risks and benefits associated with each, complicating clinical decision-making. Fourth, while incorporating registered trials and only English published trials can enhance the comprehensiveness of the evidence base, it also introduces the risk of selective reporting, especially when results from these studies remain unpublished or are only partially disclosed. Consequently, the presence of publication bias should be acknowledged as a limitation that may affect the validity of the conclusions. Finally, the inability to perform an individual patient data (IPD) analysis due to the unavailability of detailed data from study authors or sponsors represents a further limitation. Access to IPD could have allowed for more precise adjustments, subgroup analyses, and exploration of additional confounding factors, thereby enhancing the robustness of our findings. 

These limitations underscore the need for future research with longer follow-up durations, standardized outcome definitions, and access to individual patient-level data to provide a more comprehensive understanding of bexagliflozin's renal safety and efficacy.

## Conclusions

This investigation has not identified a beneficial effect of bexagliflozin on kidney disease progression, as observed with other SGLT2 inhibitors like empagliflozin, canagliflozin, and dapagliflozin. Future studies should prioritize long-term evaluations and include a broader range of patient populations, reflecting the diverse real-world settings where these medications are used. Additionally, comparative trials directly assessing bexagliflozin against established SGLT2 inhibitor agents will provide critical insights into its efficacy and safety, particularly in terms of renal outcomes. Such research will be essential for clarifying its role in clinical practice and determining whether it can match the benefits demonstrated by its counterparts. Ultimately, the use of bexagliflozin should be tailored to the patient's clinical profile and risk factors, guided by the evolving evidence in the field of SGLT2 inhibitors and considering each agent's potential advantages and limitations.

## Appendices

Appendix 1

**Table 2:** GRADE for evidence assessment. *The risk in the intervention group (and its 95% confidence interval) is based on the assumed risk in the comparison group and the relative effect of the intervention (and its 95% CI). ^a^Wide-ranging calculated prediction interval for this outcome. ^b^Wide-ranging effects in Allegretti et al. [11]. Symbols used in the GRADE summary of findings table represent the certainty of the evidence. Each filled circle (⨁) indicates one point of certainty; each empty circle (◯) represents a deducted point due to limitations such as risk of bias, inconsistency, imprecision, indirectness, or publication bias. The overall certainty is graded as follows: ⨁⨁⨁⨁: high certainty; ⨁⨁⨁◯: moderate certainty; ⨁⨁◯◯: low certainty; ⨁◯◯◯: very low certainty. GRADE Working Group grades of evidence. High certainty: we are very confident that the true effect lies close to that of the estimate of the effect. Moderate certainty: we are moderately confident in the effect estimate: the true effect is likely to be close to the estimate of the effect, but there is a possibility that it is substantially different. Low certainty: our confidence in the effect estimate is limited: the true effect may be substantially different from the estimate of the effect. Very low certainty: we have very little confidence in the effect estimate: the true effect is likely to be substantially different from the estimate of the effect. GRADE: Grading of Recommendations, Assessment, Development, and Evaluation; CI: confidence interval; MD: mean difference; RR: risk ratio; OHA: oral hypoglycemic agents; RCT: randomized controlled trial

Bexagliflozin compared to placebo or OHA for type 2 diabetic patients considering renal outcomes					
Outcomes	No. of participants (studies)	Certainty of the evidence (GRADE)	Relative effect (95% CI)	Anticipated absolute effects	
				Risk with placebo or OHA	Risk difference with bexagliflozin*
Serum creatinine	595 (2 RCTs)	⨁⨁◯◯^a,b^	-	-	MD 0.05 mmHg higher (-0.06 lower to 0.15 lower)
Systolic blood pressure	2931 (9 RCTs)	⨁⨁⨁⨁	-	-	MD 4.2 mmHg lower (5.62 lower to 2.89 lower)
Estimated glomerular filtration rate	738 (2 RCTs)	⨁⨁◯◯^a,b^	-	-	MD 0.43 mL/min/1.73 m^2^ lower (6.92 lower to 6.06 higher)
